# A synopsis of the genus *Cypholoba* Chaudoir (Coleoptera, Carabidae, Anthiini) known to occur in the Republic of South Africa

**DOI:** 10.3897/zookeys.181.2984

**Published:** 2012-04-06

**Authors:** Jonathan R. Mawdsley, Terry L. Erwin, Hendrik Sithole, Alice S. Mawdsley

**Affiliations:** 1 Department of Entomology, MRC 187, National Museum of Natural History, Smithsonian Institution, P. O. Box 37012, Washington, DC 20013-7012 USA; 2 Research Manager: Invertebrates, South African National Parks, P. O. Box 110040 Hadison Park, Kimberley South Africa; 3 Cleveland State University, 2121 Euclid Avenue, Cleveland, OH 44114 USA

**Keywords:** Identification key, distribution, savanna and woodland ecosystems, conservation

## Abstract

Nearly one third of the described species of *Cypholoba* Chaudoir (Coleoptera: Carabidae) are known to inhabit the Republic of South Africa. A key and diagnostic notes are provided for their identification, as well as notes about way of life for some of the species based on observations in the Kruger National Park. Fifteen species and subspecies of the genus are recorded from the Republic of South Africa; adult specimens of each species and subspecies are illustrated and information about the distribution of each species in the Republic of South Africa is summarized and mapped: *Cypholoba alstoni* (Péringuey), *Cypholoba alveolata* (Brême), *Cypholoba amatonga* Péringuey, *Cypholoba fritschi* (Chaudoir), *Cypholoba gracilis gracilis* (Dejean), *Cypholoba gracilis scrobiculata* (Bertoloni), *Cypholoba gracilis zuluana* Basilewsky, *Cypholoba graphipteroides graphipteroides* (Guérin-Méneville), *Cypholoba leucospilota semilaevis* (Chaudoir), *Cypholoba macilenta* (Olivier), *Cypholoba notata* (Perroud), *Cypholoba oberthueri seruana* Strohmeyer, *Cypholoba opulenta* (Boheman), *Cypholoba rutata* (Péringuey), and *Cypholoba tenuicollis aenigma* (Dohrn).

## Introduction

**Figure 1:**
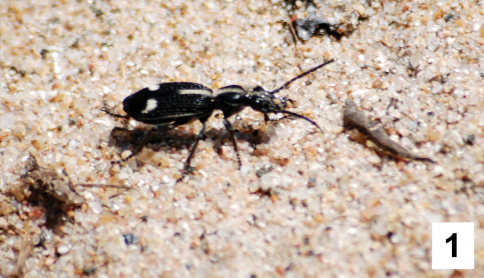
. Adult female of *Cypholoba graphipteroides graphipteroides* (Guérin-Méneville), photographed in a dry sandy streambed adjacent to the Sabie River in the Kruger National Park, RSA.

The genus *Cypholoba* Chaudoir is one of the most diverse lineages within the tribe Anthiini of the beetle family Carabidae, with 156 described species and subspecies (Lorenz 2005) distributed throughout southern and eastern Africa. These beetles are conspicuous elements of savanna and woodland ecosystems, where they are typically found running in bright sunshine over bare ground, or in short grasses (Fig. 1; Marshall and Poulton 1902). Like most other members of the tribe Anthiini, species of *Cypholoba* have the ability to excrete formic acid from their pygidial glands as a defensive behavior (Péringuey 1896). Most species in this genus are black and many species have white setal patches or setal tufts (Fig. 1) that are thought to have evolved through mimicry of Mutillidae, Formicidae, and other stinging Hymenoptera (Marshall and Poulton 1902). These beetles are of potential interest to entomologists and evolutionary biologists studying phenomena such as mimicry, aposematic coloration, and the evolution of chemical defenses. Species of *Cypholoba*, like many other Anthiini, also show close associations with particular ecosystems or vegetation communities and their activity patterns are closely tied with environmental variables such as temperature and rainfall, and overall climate patterns such as seasonal monsoons (Schmidt 2001; Mawdsley et al. 2011). Given the relatively large adult body size of most *Cypholoba* species (length 15–33 mm), their diagnostic color and setal patterns (Strohmeyer 1928) and their conspicuous activity patterns and behaviors (Schmidt 2001), these beetles could easily be incorporated into environmental monitoring programs which track overall ecosystem condition, status, and trends.

As with other Anthiini, the taxonomic history of *Cypholoba* is rather convoluted. Chaudoir (1850) initially established two genera, *Cypholoba* on p. 43 for the single species *Anthia alveolata* Brême and a second, *Polyhirma*, on p. 44 for the group of species that included *Anthia macilenta* Olivier, *Anthia gracilis* Dejean, *Anthia intermedia* Boheman, *Anthia ferretti* Reiche, *Anthia tetrastigma* Chaudoir, *Anthia leucospilota* Bertoloni, *Anthia caillaudi* Gory, and *Anthia polioloma* Chaudoir. Péringuey (1896) combined these two genera in his revision of southern African Carabidae, treating *Cypholoba* as a synonym of *Polyhirma* and recognizing 27 species-level taxa, ten of which were recorded from what is now the Republic of South Africa. Further descriptive work by other workers, primarily in the east African fauna, led to the recognition of 102 species in the genus by the time of Csiki’s (1929) comprehensive catalogue. In the “Coleopterorum Catalogus,” Csiki (1929) followed Péringuey in placing *Cypholoba* in synonymy with *Polyhirma*. Strohmeyer (1928) was the first to recognize the page priority of *Cypholoba* and also the first to attempt a comprehensive revision of this group. His revision radically altered the taxonomy of the genus, recognizing just 16 species and relegating most of the former species to subspecies status. This approach came under criticism from Basilewsky (1948, 1955) who pointed out a number of errors in Strohmeyer’s revision and argued that many of the so-called “subspecies” of Strohmeyer were separated by characteristics that suggested they were in fact perfectly good species. Basilewsky published a series of studies in which he argued against Strohmeyer’s reductionist approach while at the same time continuing to describe new species and subspecies of *Cypholoba* (Basilewsky 1948; 1955; 1963; 1967; 1980; 1983).

This paper is intended to provide an overview of the species of *Cypholoba* currently known from the Republic of South Africa (RSA). This is a fauna very much in need of good diagnostic materials, particularly keys and illustrations that can be used by non-specialists. Most of the *Cypholoba* species in RSA have never been illustrated and none of the published keys include all of the taxa now known to occur in RSA. The government of RSA has taken recent positive steps towards protecting certain carabid beetles under the South African Biodiversity Act of 2004 (Harrison and Müller, pers. comm.) and carabid beetles are increasingly being incorporated into ecosystem and agricultural monitoring programs in southern Africa (Kotze 2000; Magagula 2003). Both types of conservation approaches (carabid beetles as the subject of direct conservation efforts, and carabid beetles as environmental monitoring targets and ecological indicators) are clearly contingent on the availability of high-quality identification materials for the carabid fauna of interest. Fortunately, development of these identification materials is relatively straightforward. The species of *Cypholoba* from RSA are well represented in museum collections, as a result of large-scale survey activities that began in the 1950s and have continued more or less until the present day. The species-level taxonomy of the RSA fauna is also reasonably well known; there have been only modest changes in the taxonomy of the RSA species of *Cypholoba* since the first revision by Péringuey (1896), despite the major changes introduced by Strohmeyer (1928) in other parts of the genus. In the synopsis that follows, we follow the classification of this genus presented by Lorenz (2005) in the most recent catalogue of world Carabidae. It is hoped that this short communication will help to inspire further interest and field studies of these remarkable beetles.

## Materials and methods

We examined collections of adult *Cypholoba* Chaudoir and allied genera in the following institutional collections: Field Museum of Natural History, Chicago, Illinois (FMNH); Kruger National Park Museum (Scientific Services), Skukuza, South Africa (KNPC); South African National Collection of Insects, Pretoria, South Africa (SANC); National Museum of Natural History, Smithsonian Institution, Washington, D.C. (NMNH); Transvaal Museum, Pretoria, South Africa (TMSA). In the case of NMNH and TMSA, the specimens examined were authoritatively identified by the late P. Basilewsky, who studied this genus for many years (Basilewsky 1948; 1955; 1963; 1967; 1980; 1983).

Museum collections were augmented by a series of field visits in 2007, 2008, 2009, 2010, and 2011 to the Kruger National Park in northeastern South Africa, where adults of three species (*Cypholoba alveolata*, *Cypholoba graphipteroides*, and *Cypholoba notata*) were collected. Survey methods for *Cypholoba* species and other Anthiini involved systematic walking along roads, dry river washes, or sandy areas adjacent to major rivers. Surveys in the main park area were conducted from dawn until dusk and under all available weather conditions. Head-lamping surveys were conducted at night for Anthiini and other Carabidae in the N’waswitshaka Research Camp at Skukuza. Pitfall traps were also widely deployed throughout the Skukuza Ranger District of the Kruger National Park, to study the distribution of species of Carabidae across different vegetation communities and to record the responses of carabid assemblages to common landscape-scale disturbances such as mammalian grazing and ground fire. Driving surveys (Mawdsley et al. 2011) were also employed as a survey technique for larger Anthiini, as well as species of *Tefflus* Leach (Coleoptera: Carabidae: Panagaeini); adults of larger species of *Cypholoba* such as *Cypholoba graphipteroides* can be detected during driving surveys. When encountered during diurnal surveys, the individual beetles were highly conspicuous and could be easily captured by hand. Further information about our survey methods is available in papers by Mawdsley and Sithole (2008; 2009), Mawdsley (2009; 2011), and Mawdsley et al. (2011).

Methods and species concepts follow those previously described (Erwin and Kavanaugh 1981; 1991). The species validation and diagnosis format follows as closely as possible that suggested in Erwin and Johnson (2000). Measurements of length (ABL) and width (TW) follow those of Ball (1972) and Kavanaugh (1979): ABL (apparent body length), measured from apex of labrum to apex of longer elytron.

### Cypholoba

Chaudoir, 1850

http://species-id.net/wiki/Cypholoba

Cypholoba Chaudoir, 1850:43, type species *Cypholoba alveolata* Brême, 1844:293
Polyhirma Chaudoir, 1850:44; synonymy by Strohmeyer (1928:298)
Diabatus Gistel 1857:36; synonymy by Csiki (1929:381)

#### Diagnosis.

Medium to large Anthiini (length 15–33 mm) with the following combination of adult attributes: head with distinct basal constriction or “neck;” second labial palpomere distinctly longer than third; pronotum rectangular or cordiform (heart-shaped), not at all markedly elevated or swollen, lacking lateral or basal flanges; elytron with rows of elevated costae which alternate with rows of large, deep punctures (in most species); elytral punctures with brown, yellow, or orange setae at bottom in many species; rows of punctures and setae not extending to apex of elytron in most species; apex of elytron flat or planate in most species; elytron often with patterned setae and/or pubescence; posterior margin of elytron usually obliquely truncate; elytron lacking an apical spine.

#### Distribution.

Southern and eastern Africa.

#### Included species in the Republic of South Africa.

The species list below and the arrangement of descriptions that follow are ordered alphabetically.

*Cypholoba alstoni* (Péringuey), Botswana, Namibia, Republic of South Africa (RSA)

*Cypholoba alveolata* (Brême), Botswana, RSA, Zimbabwe

*Cypholoba amatonga* Péringuey, Mozambique, RSA

*Cypholoba fritschi* (Chaudoir), Botswana, Namibia, RSA, Zambia

*Cypholoba gracilis gracilis* (Dejean), Botswana, Mozambique, Namibia, RSA

*Cypholoba gracilis scrobiculata* (Bertoloni), Mozambique, RSA

*Cypholoba gracilis zuluana* Basilewsky, RSA

*Cypholoba graphipteroides graphipteroides* (Guérin-Méneville), Mozambique, RSA, Zimbabwe

*Cypholoba leucospilota semilaevis* (Chaudoir), Mozambique, RSA

*Cypholoba macilenta* (Olivier), Lesotho, RSA, Zimbabwe

*Cypholoba notata* (Perroud), RSA, Swaziland

*Cypholoba oberthueri seruana* Strohmeyer, Botswana, RSA

*Cypholoba opulenta* (Boheman), Namibia, RSA

*Cypholoba rutata* (Péringuey), Mozambique, RSA, Zimbabwe

*Cypholoba tenuicollis aenigma* (Dohrn), Botswana, RSA, Zambia, Zimbabwe

#### Key to adults of *Cypholoba* Chaudoir species from the Republic of South Africa.

**Table:** 

1	Elytron without white or yellow patches or linear bands of setae in apical half; basal half of elytron along suture at base with at most a narrow white or yellow patch or band of setae	2
–	Elytron with distinct white or yellow patches or linear bands of setae in apical half; basal half of elytron typically also with basal patches of white or yellow setae	10
2	Pronotum elongate, slender, spindle-shaped	3
–	Pronotum broader, either rounded, rectangular, or heart-shaped	5
3	Pronotum with well-defined longitudinal median impression; elytral punctures round	*Cypholoba gracilis gracilis* (Dejean)
–	Pronotum with median line feebly impressed at base and otherwise not at all impressed along most of length, sometimes with a narrow raised ridge; elytral punctures oval	4
4	Pronotum median line with a round impression at base and becoming obsolete on disc	*Cypholoba gracilis scrobiculata* (Bertoloni)
–	Pronotum median line with a narrow impression at base and a narrow, slightly raised ridge on disc	*Cypholoba gracilis zuluana* Basilewsky
5	Elytral punctures large, those on disc with orange-red setae deep within each puncture	6
–	Elytral punctures small, those on disc without orange-red setae deep within each puncture	7
6	Apparent body length (ABL) 24–28 mm; pronotum elongate and somewhat heart-shaped, width of pronotum less than width of eyes; elytra with six costae and large alveolate punctures	*Cypholoba amatonga* Péringuey
	Apparent body length (ABL) 31–33 mm; pronotum broader and distinctly heart-shaped, width of pronotum distinctly greater than width of eyes; elytra with six costae, fifth costa as measured from lateral margin short, only reaching to basal sixth of elytra; elytral punctures large, alveolate	*Cypholoba alveolata* (Brême)
7	Elytra and pronotum without patterned pubescence or setae of any kind; elytral costae and rows of punctures continuing to apex	*Cypholoba alstoni* (Péringuey)
–	Elytra and often pronotum with small setal tufts or patches at base; elytral costae and rows of punctures usually becoming obsolete on disc, apex of elytra smooth	8
8	Pronotal base, scutellum and base of elytra each with a small white or yellow setal tuft, barely noticeable; elytral costae broad, smooth, and shining, becoming obsolete at apical third; apical third of elytra shining	*Cypholoba fritschi* (Chaudoir)
–	Pronotum and/or elytra with more extensive setal patches; elytral costae narrow; apical third of elytra matte, not shining	9
9	Elytral surface markedly rugose, each elytron with a narrow linear band of yellow setae along suture on basal third	*Cypholoba rutata* (Péringuey)
–	Elytral punctures very small, costae present but not markedly elevated, elytral surface largely smooth; each elytron with an ovate or rectangular patch of yellow setae adjacent to suture on basal fifth	*Cypholoba opulenta* (Boheman)
10	Elytra with patches of grey or white setae on disc at apical third or apical fourth	11
–	Elytra with patches of pale setae at base and/or apex of elytra only, adjacent to the suture	13
11	Apparent body length (ABL) 15-17 mm; pronotum elongate, slender, spindle-shaped; each elytron with a second transverse band of white setae at mid-elytron	*Cypholoba tenuicollis aenigma* (Dohrn)
–	Apparent body length (ABL) 24-28 mm; pronotum broader, heart-shaped; each elytron with a patch of setae at apical third and a narrow line of white setae along the suture on the basal third of elytra; no second band of white setae at mid-elytron	12
12	Pronotal and elytral setae uniformly greyish-white; a narrow linear band of setae along midline of pronotum, a narrow linear band of setae along elytral suture on basal two-fifths, and a pair of arcuate patches located at apical third	*Cypholoba graphipteroides graphipteroides* (Guérin-Méneville)
–	Pronotal setae and the setal band at the base of the elytra yellowish orange; elytral setal patch placement similar to above, except for coloration, discal patches bright white	*Cypholoba leucospilota semilaevis* (Chaudoir)
13	Elytra with apical third smooth, shining, except for apical patch of white setae; basal two-thirds of elytra with rows of costae and large punctures, which lack reddish-orange pubescence	*Cypholoba notata* (Perroud)
–	Elytra with apical fourth or fifth smooth, shining, except for apical patch of white setae; basal three-quarters of elytra with rows of costae and large punctures, reddish-orange pubescence present deep inside each puncture	14
14	Elytra with apical fourth smooth, shining, except for apical patch of white setae; each elytron with seven costae, of which the sixth is very short	*Cypholoba macilenta* (Olivier)
–	Elytra with apical fifth smooth, shining, except for apical patch of white setae; each elytron with seven costae subequal in size	*Cypholoba oberthueri seruana* Strohmeyer

#### Cypholoba alstoni

(Péringuey, 1892a)

http://species-id.net/wiki/Cypholoba_alstoni

Figs 2 29

Polyhirma alstoni Péringuey (1892a:14; type locality “British Bechuanaland,” syntype series in South African Museum, Cape Town)
Cypholoba alstoni (Péringuey) Lorenz (2005:513)

##### Diagnosis.

Apparent body length (ABL) 23-24 mm; easily separated from all other species of *Cypholoba* in RSA by the lack of patterned pubescence on the elytra and the continuation of the rows of elytral punctures and costae until the elytral apices. The sympatric species *Cypholoba fritschi* is similar but is larger in ABL (25-31 mm in length) and its elytral costae become obsolete on the disc shortly after mid-elytron, leaving the apical third of the elytra almost entirely smooth.

**Figures 2–15.**
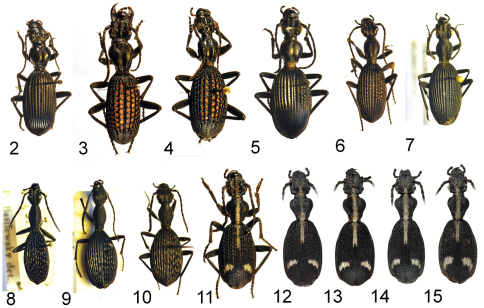
Adult specimens of *Cypholoba* species, from the TMSA collection unless otherwise indicated. **2** *Cypholoba alstoni* (Péringuey), male, Niekerk’s Hope in Griqualand West, Northern Cape Province, RSA **3** *Cypholoba alveolata* (Brême), male, Warmbath, Limpopo Province, RSA **4** *Cypholoba amatonga* Péringuey, male, Soutpansberg, Limpopo Province, RSA **5** *Cypholoba fritschi* (Chaudoir), male, Twee Rivieren, Northern Cape Province, RSA **6** *Cypholoba gracilis gracilis* (Dejean), male, Zoutpan, Gauteng Province, RSA **7** *Cypholoba gracilis gracilis* (Dejean), female, Vanwyksfontein Farm, Northern Cape Province, RSA **8** *Cypholoba gracilis scrobiculata* (Bertoloni), male, Zoutpansberg, Limpopo Province, RSA **9** *Cypholoba gracilis scrobiculata* (Bertoloni), female, Inhambane, Mozambique **10** *Cypholoba gracilis zuluana* Basilewsky, male, “E. Zululand,” KwaZulu/Natal Province, RSA **11** *Cypholoba graphipteroides graphipteroides* (Guérin-Méneville), male, 20-26 km NE of Pietersburg, Limpopo Province, RSA **12–15** *Cypholoba graphipteroides graphipteroides* collected at sites along the Sabie River west of Paul Kruger Gate in the Kruger National Park, showing intrapopulational variation in elytral setal patterns (NMNH collection).

##### Materials examined.

107 specimens from the following localities: RSA: Northern Cape Province: Farm Brulpan, Marydale, Mata Mata, Niekerk’s Hope in Griqualand West, 30 km E Pofadder, 46 km N Pofadder, Tswalu Nature Reserve, Twee Rivieren.

#### Cypholoba alveolata

(Brême, 1844)

http://species-id.net/wiki/Cypholoba_alveolata

Figs 3 25 29 34

Anthia alveolata Brême (1844:293, pl. 7 f. 5, type locality “Port-Natal,” holotype in Museum National d’Histoire Naturelle, Paris)
Polyhirma alveolata (Brême) Péringuey (1896:351)
Cypholoba caillaudi alveolata (Brême) Strohmeyer (1928:322)
Cypholoba alveolata (Brême) Strohmeyer (1928:448)

##### Diagnosis.

Apparent body length (ABL) 31–33 mm; one of the largest species of *Cypholoba* in RSA and the largest species that has the distinctive alveolate elytral punctures with reddish-orange pubescence at the bottom of the punctures (see Fig. 3). It can be separated from *Cypholoba macilenta*, *Cypholoba notata*, and *Cypholoba oberthueri seruana* by the lack of a white patch of setae across both elytral apices and from *Cypholoba amatonga* by its larger ABL (24–28 mm length in *Cypholoba amatonga*) and the differences in elytral surface sculpture mentioned in the key above. Some specimens have a patch of white setae on each elytron immediately adjacent to the scutellum, while others also have a patch of white setae on the scutellum and/or a line of white setae along the midline of the pronotum.

##### Materials examined.

159 specimens from the following localities: RSA: Gauteng Province: Johannesburg, Moloto, Pienaars River Dam, Pretoria, Rosslyn, Zoutpan. KwaZulu/Natal Province: Duku duku, Durban, Ndumu, Pongola River, St. Lucia Bay, Umfolozi, Waterval, “E Zululand.” Limpopo Province: Bandelierkop, 25 km NE Ellisras, Farm Scrutton, Haenertsburg, Klein Letaba, 18 miles W Letaba Rest Camp, Letaba Rest Camp in Kruger National Park, Marblehall, Messina, Mokeetse, Pietersburg, 22 miles E Pietersburg, 20-26 km N Pietersburg, Potgietersrus, Sedula near Leydsdorp, Shilouvane, Warmbath, Woodbush. Mpumalanga Province: Barberton, 3 km NW Barberton, Grootdraai, Lydenburg, Nelspruit, Nkuhlu Plots in Kruger National Park, Sabie River road west of Paul Kruger Gate, Skukuza. North West Province: Marico River, Rustenburg, Vryberg. Province not specified: “Transvaal.”

##### Notes on biology.

During our surveys (see Materials and Methods above), adults of this species were found in riverine and upland areas of the Kruger National Park. Specific vegetation communities (Gertenbach 1983) where adults were collected included riverine gallery forest and upland *Acacia nigrescens* Olivier *- Combretum apiculatum* Sonder savanna. Adults were collected diurnally, in pitfall traps, and with headlamps at night.

**Figures 16–28.**
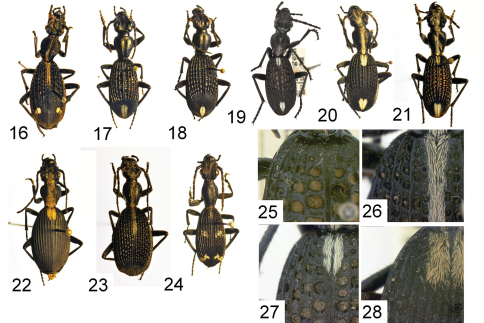
Adult specimens and diagnostic features of *Cypholoba* species, from the TMSA collection unless otherwise indicated **16** *Cypholoba leucospilota semilaevis* (Chaudoir), male, “E. Zululand,” KwaZulu/Natal Province, RSA **17** *Cypholoba macilenta* (Olivier), male, Groenkloof, Gauteng Province, RSA **18** *Cypholoba notata* (Perroud), male, Shilouvane, Limpopo Province, RSA **19** *Cypholoba notata* (Perroud), male, Empangeni, KwaZulu/Natal Province, RSA (NMNH collection) **20** *Cypholoba notata* (Perroud), male, Pongola River, KwaZulu/Natal Province, RSA **21** *Cypholoba oberthueri seruana* Strohmeyer, male, Allemanskraal, Free State Province, RSA **22** *Cypholoba opulenta* (Boheman), male, Us Pass, Khomas Hochland, Namibia **23** *Cypholoba rutata* (Péringuey), male, Pafuri, Kruger National Park, Limpopo Province, RSA **24** *Cypholoba tenuicollis aenigma* (Dohrn), Zimbabwe **25** base of elytra showing punctures, *Cypholoba alveolata* **26** base of elytra showing punctures, *Cypholoba graphipteroides graphipteroides* **27** base of elytra showing punctures, *Cypholoba notata* **28** base of elytra showing punctures, *Cypholoba opulenta*.

**Figures 29–32.**
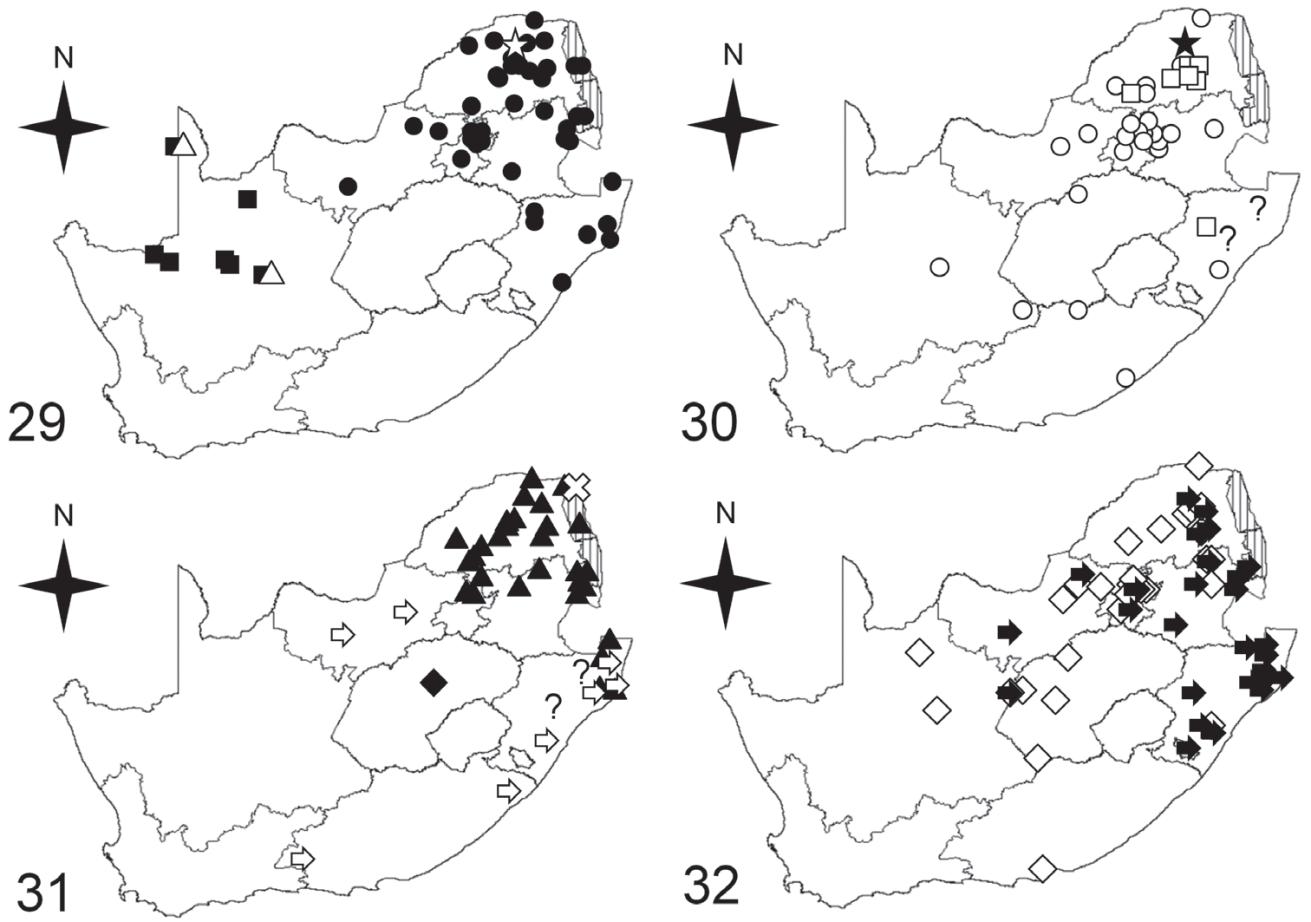
Maps showing distributions of *Cypholoba* species in RSA **29** black squares for *Cypholoba alstoni*, black circles for *Cypholoba alveolata*, white star for *Cypholoba amatonga*, white triangles for *Cypholoba fritschi* **30** white circles for *Cypholoba gracilis gracilis*, black star for *Cypholoba gracilis scrobiculata*, question marks (indeterminate localities) for *Cypholoba gracilis zuluana*, white squares for *Cypholoba tenuicollis aenigma* **31** black triangles for *Cypholoba graphipteroides graphipteroides*, question marks (indeterminate localities) for *Cypholoba leucospilota semilaevis*, black diamond for *Cypholoba oberthueri seruana*, white arrow for *Cypholoba opulenta*, white X for *Cypholoba rutata* **32** white diamonds for *Cypholoba macilenta*, black arrows for *Cypholoba notata*.

#### Cypholoba amatonga

Péringuey, 1892b

http://species-id.net/wiki/Cypholoba_amatonga

Figs 4 29

Cypholoba amatonga Péringuey (1892:102b, type locality “Amatongaland, Delagoa Bay,” syntype series in South African Museum, Cape Town)
Polyhirma amatonga (Péringuey) Péringuey (1896:350)
Cypholoba chaudoiri amatonga (Péringuey) Strohmeyer (1928:321)
Cypholoba amatonga (Péringuey) Strohmeyer (1928:448)

##### Diagnosis.

Apparent body length (ABL) 24-28 mm; similar to *Cypholoba alveolata* but separated from that species by the smaller ABL (31-33 mm in *Cypholoba alveolata*) and by the differences in elytral surface sculpture mentioned in the key above.

##### Materials examined. 

2 specimens from the following locality: RSA: Limpopo Province: Soutpansberg.

#### Cypholoba fritschi

(Chaudoir, 1883)

http://species-id.net/wiki/Cypholoba_fritschi

Figs 5 29

Polyhirma fritschi Chaudoir (1883:27, type locality “Kuruman,” holotype in Museum National d’Histoire Naturelle, Paris)
Cypholoba opulenta fritschi (Chaudoir) Strohmeyer (1928:368)
Cypholoba fritschi (Chaudoir) Strohmeyer (1928:450)

##### Diagnosis.

Apparent body length (ABL) 25–31 mm; distinctive for its large body size and for the lack of patterned pubescence or setae on the elytra, although some specimens may have a small patch of yellow setae adjacent to the scutellum. As noted by Péringuey (1896), there is also a tuft of pubescence on the scutellum. The elytral surface sculpture is also diagnostic, with the rows of costae becoming obsolete shortly after mid-elytron and the apical third smooth and strongly shining.

##### Materials examined.

11 specimens from the following localities: RSA: Northern Cape Province: Niekerk’s Hope in Griqualand West, Twee Rivieren.

##### Notes on taxonomy.

Strohmeyer (1928) had placed this species as a subspecies of *Cypholoba opulenta* but as pointed out by Basilewsky (1948) this is clearly an error as the two species are quite distinct and have different proportions, vestiture, and surface sculpture.

#### Cypholoba gracilis gracilis

(Dejean, 1831)

http://species-id.net/wiki/Cypholoba_gracilis_gracilis

Figs 6, 7 30

Anthia gracilis Dejean (1831:468, type locality “cap de Bonne-Espérance,” holotype in Museum National d’Histoire Naturelle, Paris).
Polyhirma gracilis (Dejean) Péringuey (1896:342-343)
Cypholoba gracilis gracilis (Dejean) Strohmeyer (1928:303)

##### Diagnosis.

Apparent body length (ABL) 17-20 mm; easily recognized by its slender body form, spindle-shaped pronotum, and lack of white setae or pubescence on the elytra. The shape and size of the elytral punctures (described in the key) will separate this subspecies from both *Cypholoba gracilis scrobiculata* and *Cypholoba gracilis zuluana*. In both *Cypholoba gracilis gracilis* and *Cypholoba gracilis scrobiculata* the shape of the elytra is sexually dimorphic and thus both forms are illustrated here.

##### Materials examined.

35 specimens from the following localities: RSA: Eastern Cape Province: Aliwal North, Dorset. Free State Province: Bothaville. Gauteng Province: 11 km SE Bronkhorstspruit, Bronkhorstspruit, Brooklyn, Florida, Pretoria, Roodeplaat, Rosslyn, Welgedacht, Zoutpan, Zusterstroom. KwaZulu/Natal Province: Waterval. Limpopo Province: Messina, Moordrift, Naboomspruit, Nylsvley, Pietersburg, Rhenosterpoort, Smith Farm. Mpumalanga Province: Argent, 14 miles E Middelburg, Middelburg, Moloto, Waterval Onder. Northern Cape Province: Niekerk’s Hope in Griqualand West, Vanwyksfontein Farm. North West Province: Lichtenburg, Rustenburg.

#### Cypholoba gracilis scrobiculata

(Bertoloni, 1847)

http://species-id.net/wiki/Cypholoba_gracilis_scrobiculata

Figs 8, 9 30

Anthia scrobiculata Bertoloni (1847:90, type locality “in provincia Inhambanensi Monzambici,” holotype in Accademia della Scienze dell’Istituto Bologna, Italy)
Polyhirma scrobiculata (Bertoloni) Péringuey (1896:342)
Cypholoba gracilis scrobiculata (Bertoloni) Strohmeyer (1928:315)

##### Diagnosis.

Apparent body length (ABL) 19–20 mm; similar in general body form and appearance to *Cypholoba gracilis gracilis* but differing from that subspecies in having the median line of the pronotum not markedly impressed and in having the elytral punctures oval rather than round.

##### Materials examined.

1 specimen from the following locality: RSA: Limpopo Province: Zoutpansberg.

##### Notes on taxonomy.

Based on the rather significant differences in the pronotal and elytral surface sculpture, *Cypholoba gracilis scrobiculata* may ultimately prove to represent a species distinct from *Cypholoba gracilis*. A thorough review of the *Cypholoba gracilis* species complex with a rigorous evaluation of the 41 subspecific taxa proposed by Strohmeyer (1928) is needed.

#### Cypholoba gracilis zuluana

Basilewsky, 1948

http://species-id.net/wiki/Cypholoba_gracilis_zuluana

Figs 10 30

Cypholoba gracilis zuluana Basilewsky (1948:111, type locality “Zululand,” syntype series in Naturhistoriska Riksmuseet, Stockholm)

##### Diagnosis.

Apparent body length (ABL) 19–20 mm; similar in general body form and appearance to *Cypholoba gracilis gracilis* and *Cypholoba gracilis scrobiculata* but differing from those two subspecies in the pronotal surface sculpturing, as noted in the key. There are also subtle differences in the arrangement and size of the elytral punctures and elytral costae between all three subspecies (see Figs 6–10).

##### Materials examined.

6 specimens from the following localities: RSA: KwaZulu/Natal Province: “E. Zululand,” “Zululand.”

#### Cypholoba graphipteroides graphipteroides

(Guérin-Méneville, 1845)

http://species-id.net/wiki/Cypholoba_graphipteroides_graphipteroides

Figs 1 11, 12, 13, 14, 15 26 31 33

Anthia graphipteroides Guérin-Méneville (1845:285, type locality “in regione Massilicatzi,” holotype in Museum National d’Histoire Naturelle, Paris)
Polyhirma graphipteroides (Guérin-Méneville) Péringuey (1896:346)
Cypholoba graphipteroides graphipteroides (Guérin-Méneville) Strohmeyer (1928:343)

##### Diagnosis.

Apparent body length (ABL) 24–27 mm; this species is easily separated from most of the other sympatric species of *Cypholoba* by the distinctive pattern of pubescence and setae on the elytra (Fig. 11). Adults could potentially be confused with *Cypholoba leucospilota*, but in that species the pubescence on the pronotum and elytral suture is yellowish and the patches of setae at apical third are brilliant white (Fig. 16).

##### Materials examined.

77 specimens from the following localities: RSA: Gauteng Province: Pretoria, Rosslyn. KwaZulu/Natal Province: Hluhluwe, Mkuze Game Reserve, Ndumu, St. Lucia Bay. Limpopo Province: Alma, Koedoesrivier, Letaba Rest Camp in the Kruger National Park, 9–14 miles E Louis Trichardt, 75 km W Messina, Nylstroom, Nylsvley, Pietersburg, 9 miles N Pietersburg, 20–26 miles NE Pietersburg, Potgietersrus, Punda Maria Rest Camp in the Kruger National Park, Rust de Winter, Shilouvane, Thabina, Warmbath. Mpumalanga Province: Kaapmuiden, 20 km SW Kaapmuiden, Louws Creek, Lydenburg, N’waswitshaka Research Camp, Pretoriuskop, Sabie river banks west of Paul Kruger Gate, Skukuza. Province uncertain: Lebombo Mountains.

##### Notes on biology.

This is easily the most abundant and frequently encountered species of Anthiini in the Kruger National Park, RSA. Adults (Fig. 1) emerge in the early rainy season (November-December) and can be locally abundant. Adults are active both nocturnally and diurnally and are typically found in riverine or riparian areas near flowing water. The charactistic behavior observed in this species, as with most Anthiini, is a rapid walking behavior which appears to be associated primarily with foraging but also is likely involved in searching for conspecifics and prospective larval habitats. We have observed adults of this species in several distinct vegetation communities (riverine gallery forest, open *Combretum apiculatum* – *Acacia nigrescens* savanna, *Phragmites* reed beds) and microhabitats (sand or dirt roads adjacent to riverine communities, dry sand wash, and riverine sand bars). This species also occasionally enters human-inhabited areas; we have found individuals in the N’waswitshaka Research Camp at Skukuza and also in the main tourist areas at the Skukuza tourist camp. Marshall and Poulton (1902) suggest that this species may be a mimic of Mutillidae and other stinging Hymenoptera. Our observations suggest that, while there are certainly similarities in color pattern and behavior between adults of *Cypholoba graphipteroides* and sympatric Mutillidae, adults of *Cypholoba graphipteroides* are actually significantly larger in size than most of the sympatric black-and-white mutillid wasps, rendering the resemblance less than exact. We infer from these observations that selection pressures may not be operating as intensely on this species as on other Carabidae (for examples of carabid beetles which are much more convincing mimics of Mutillidae, see Marshall and Poulton 1902).

**Figure 33.**
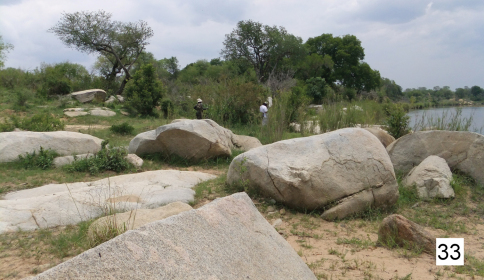
Riverine/riparian area along the south bank of the Sabie River west of Paul Kruger Gate in the Kruger National Park, RSA. A collecting site for *Cypholoba graphipteroides graphipteroides*.

**Figure 34.**
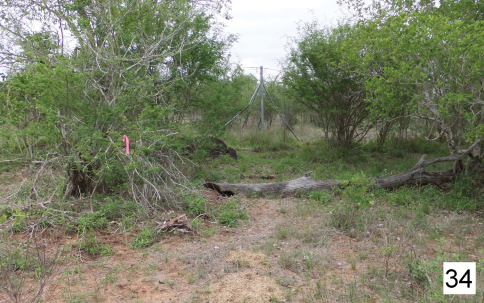
Upland *Acacia nigrescens* – *Combretum apiculatum* savanna near Skukuza in the Kruger National Park, RSA. A collecting site for *Cypholoba alveolata*.

##### Notes on taxonomy.

Strohmeyer (1928) recognized 20 subspecies in *Cypholoba graphipteroides*, many of which were separated on the basis of differences in the setal patterns of the elytra. Many of these taxa are doubtfully distinct from the nominate form and the whole group is in need of a careful revision. Such a revision should include examination of extensive series in order to determine the extent of intrapopulational variation in the elytral setal patterns. Figures 12–15 illustrate the variation in elytral setal patterns within a single population of this species, located west of Paul Kruger Gate along the Sabie River in the Kruger National Park.

#### Cypholoba leucospilota semilaevis

(Chaudoir, 1861)

http://species-id.net/wiki/Cypholoba_leucospilota_semilaevis

Figs 16 31

Polyhirma semilaevis Chaudoir (1861:571–572, type locality “de la baie Delagoa,” syntype series in Museum National d’Histoire Naturelle, Paris)
Polyhirma semilevis Péringuey (1896:344–345), unjustified emendation
Cypholoba leucospilota semilaevis (Chaudoir) Strohmeyer (1928:351)

##### Diagnosis.

Apparent body length (ABL) 22–28 mm; similar in general appearance to *Cypholoba graphipteroides* but distinguished from that species by the color of the pronotal and elytral pubescence and setae. The bands of pubescence on the pronotum and elytral base are yellow in *Cypholoba leucospilota* (white or grey in *Cypholoba graphipteroides*), and the bands of pubescence on the apical third of the elytra are a much brighter white in *Cypholoba leucospilota* than in *Cypholoba graphipteroides*.

##### Materials examined.

5 specimens from the following localities: RSA: KwaZulu/Natal Province: “E. Zululand,” “Zululand.”

#### Cypholoba macilenta

(Olivier, 1795)

http://species-id.net/wiki/Cypholoba_macilenta

Figs 17 32

Carabus macilentus Olivier (1795:26, pl. 11 f. 130, type locality “Cap de Bonne-Espérance,” holotype in Museum National d’Histoire Naturelle, Paris)
Polyhirma macilenta (Olivier) Péringuey (1896:348)
Cypholoba macilenta (Olivier) Strohmeyer (1928:335)

##### Diagnosis.

Apparent body length (ABL) 20–23 mm; similar to *Cypholoba notata* and *Cypholoba oberthueri* but differing from both species in the length of the elytral costae. The elytral costae are roughly equal in length in *Cypholoba macilenta* (see Fig. 17) while the lateral costae are distinctly longer than those on the disc in *Cypholoba notata* and *Cypholoba oberthueri* (see Figs 18–21). Adults could potentially also be confused with *Cypholoba amatonga* but that species lacks the patch of white pubescence at the apex of the elytra and the apical portion of the elytra is more rugosely punctate, not flat and strongly shining as in *Cypholoba macilenta*.

##### Materials examined.

127 specimens from the following localities: RSA: Eastern Cape Province: De la Rey. Free State Province: Boshof, Bothaville, H. F. Verwoerd Dam, Krugersdrift Dam. Gauteng Province: Bronkhorstspruit, Groenkloof, Johannesburg, Moloto, Muldersdrift, Pretoria, Randfontein, Rosslyn, Valhalla, Zoutpan. KwaZulu/Natal Province: Waterval. Limpopo Province: Messina, Moordrift, Pienaars River, Pietersburg, 20–26 km NE Pietersburg, Shilouvane, Woodbush. Mpumalanga Province: Lydenburg, Middelburg, 14 miles E Middelburg, Waterval Onder. Northern Cape Province: Kimberley, Niekerk’s Hope in Griqualand West, Vanwyksfontein Farm. North West Province: Lichtenburg, Marico, Rustenburg.

#### Cypholoba notata

(Perroud, 1846)

http://species-id.net/wiki/Cypholoba_notata

Figs 18, 19, 20, 27 32

Anthia notata Perroud (1846:50–56, type locality “de l’ intérieur de Natal,” syntypes in Museum National d’Histoire Naturelle, Paris)
Polyhirma notata (Perroud) Péringuey (1896:348)
Cypholoba semisuturata notata (Perroud) Strohmeyer (1928:337)
Cypholoba notata (Perroud) Strohmeyer (1928:452)

##### Diagnosis.

Apparent body length (ABL) 21–23 mm; elytral surface sculpture distinctive with the entire apical third lacking the rows of punctures and costae found on the more basal portions of the elytra. The lateral elytral costae are longer than the costae on mid-disc or the suture. The patterns of pubescence vary in this species; some specimens possess white linear bands of setae on the head, pronotum, and base of the elytra while other specimens lack these bands (Figs 14, 15). We have also examined highly abraded specimens that lack all of the elytral and pronotal setae but in these specimens the elytral surface sculpturing is still diagnostic.

##### Materials examined.

96 specimens from the following localities: RSA: Gauteng Province: Malvern, Pretoria. KwaZulu/Natal Province: Empangeni, 30 miles N of Empangeni, Hluhluwe, Maritzburg, Mkuze, “Natal,” Ntambanana, Pinetown, Pongola, Pongola River, St. Lucia Bay, Thorny Bush, Ubombo Mountains, Weenen, “Zululand,” “E. Zululand.” Limpopo Province: Koedoes Rivier, Sedula near Leydsdorp, Shilouvane, Thabina, Zoutpansberg. Mpumalanga Province: Barberton, Grootdraai, Lydenburg, Nelspruit, Sabie River bank west of Paul Kruger Gate, Waterval River Pass. Northern Cape Province: Kimberley. North West Province: Marico River, Vryburg.

##### Notes on biology.

We observed adults of this species running along dirt roads in bright sunshine through open *Combretum apiculatum* – *Acacia nigrescens* savanna in close proximity to the Sabie River in the Kruger National Park, RSA.

#### Cypholoba oberthueri seruana

Strohmeyer, 1928

http://species-id.net/wiki/Cypholoba_oberthueri_seruana

Figs 21 31

Cypholoba semisuturata seruana Strohmeyer (1928:337, type locality “Ost-Betschuanaland,” one syntype from “Sogosse” the other syntype from “Serue” in Museum für Naturkunde, Berlin)

##### Diagnosis.

Apparent body length (ABL) 21–24 mm; similar in appearance to *Cypholoba macilenta* and *Cypholoba notata* but differing from both species in the length of the elytral costae (as noted in the key) and the elytral proportions which are somewhat longer and narrower in *Cypholoba oberthueri seruana* than in the other two species. This is apparently a rare form, at least in collections; it is known at present from two localities in Botswana and the nominate subspecies is known only from Zimbabwe (Strohmeyer 1928).

##### Materials examined.

1 specimen from the following locality: RSA: Free State, Allemanskraal.

#### Cypholoba opulenta

(Boheman, 1860)

http://species-id.net/wiki/Cypholoba_opulenta

Figs 22, 28 31

Polyhirma opulenta Boheman (1860:9, type locality “juxta fluvium Svakop,” holotype in Naturhistoriska Riksmuseet, Stockholm)
Cypholoba opulenta (Boheman) Strohmeyer (1928:367)

##### Diagnosis.

Apparent body length (ABL) 18–24 mm; easily recognized by the golden-yellow pubescence on the head, pronotum, and base of elytra adjacent to the suture (Fig. 22). The elytral surface sculpturing is also diagnostic, with small narrow costae separated by flat intervals with a single row of minute punctures in each interval (Fig. 28).

##### Materials examined.

45 specimens from the following localities: Eastern Cape Province: Nduma, Willowmore. KwaZulu/Natal Province: Maritzburg, Melmoth, Mkuze, St. Lucia, “E. Zululand.” North West Province: Lichtenburg, 30 km W Vryberg.

#### Cypholoba rutata

(Péringuey, 1892b)

http://species-id.net/wiki/Cypholoba_rutata

Figs 23 31

Polyhirma rutata Péringuey (1892b:101, type locality “Zambeze,” syntype series in South African Museum, Cape Town)
Cypholoba divisa rutata (Péringuey) Strohmeyer (1928:359–360)
Cypholoba rutata (Peringuey) Strohmeyer (1928:453)

##### Diagnosis.

Apparent body length (ABL) 22–27 mm; similar in body proportions and surface sculpturing to *Cypholoba graphipteroides* and *Cypholoba leucospilota* but lacking the oblique bands of white setae and pubescence that are found at the apical third of the elytra in those species (see Figs 11–16).

##### Materials examined.

1 specimen from the following locality: RSA: Limpopo Provice: Pafuri in Kruger National Park.

#### Cypholoba tenuicollis aenigma

(Dohrn, 1881)

http://species-id.net/wiki/Cypholoba_tenuicollis_aenigma

Figs 24 30

Anthia aenigma Dohrn (1881:326, type locality not indicated, type material originally in Natural History Museum, Stettin, and apparently destroyed in World War II)
Polyhirma aenigma (Dohrn) Péringuey (1896:343-344, pl. 4 f. 6)
Cypholoba gracilis aenigma (Dohrn) Strohmeyer (1928:303)
Cypholoba aenigma (Dohrn) Strohmeyer (1928:447)

##### Diagnosis.

Apparent body length (ABL) 15–17 mm; similar in size, body proportions, and surface sculpturing to smaller individuals of *Cypholoba gracilis* but easily separated from that species by its patterned elytral pubescence (Fig. 19) and smaller body size (adults of all subspecies of *Cypholoba gracilis* are 17–22 mm in ABL). Adults are also superficially similar to those of species in the genus *Eccoptoptera* Chaudoir but in species of that genus the pronotum is broadly rounded and markedly convex (Péringuey 1896; Strohmeyer 1928).

##### Materials examined.

84 specimens from the following localities: RSA: KwaZulu/Natal Province: Entabeni. Limpopo Province: Haenertsburg, Moddernek, Pietersburg, Shilouvane, Woodbush, Wylie’s Poort.

## Supplementary Material

XML Treatment for Cypholoba
